# Sjögren's syndrome concurrent with protein‐losing gastroenteropathy with secondary systemic capillary leak syndrome : A case report

**DOI:** 10.1002/ccr3.1675

**Published:** 2018-07-31

**Authors:** Kei Watanabe, Shinichiro Murakami, Masahiro Misago, Mai Yoshikawa, Daisuke Tamai, Shinichiro Nakao, Takato Ueoka, Mototoshi Ito, Yasuhisa Shinomura, Nobuyuki Kajiwara

**Affiliations:** ^1^ Department of Emergency and General Medicine Ikeda City Hospital Ikeda Osaka Japan; ^2^ Department of Gastroenterology Ikeda City Hospital Ikeda Osaka Japan; ^3^ Department of Nephrology Ikeda City Hospital Ikeda Osaka Japan

**Keywords:** gamma globulin therapy, protein‐losing gastroenteropathy, Sjögren's syndrome, steroid therapy, systemic capillary leak syndrome

## Abstract

Sjögren's syndrome concurrent with protein‐losing gastroenteropathy can develop into secondary systemic capillary leak syndrome. Thus, it is important to diagnose the condition as soon as possible and simultaneously administer treatment for Sjögren's syndrome, protein‐losing gastroenteropathy, and systemic capillary leak syndrome.

## 1 INTRODUCTION

Protein‐losing gastroenteropathy is a rare condition. In some cases, it has been found to coexist with connective tissue diseases, such as Sjögren's syndrome.^1^ We experienced the case of a patient with Sjögren's syndrome concurrent with protein‐losing gastroenteropathy who subsequently developed secondary systemic capillary leak syndrome, making their condition temporarily critical. However, the combination of various treatments resulted in an improvement. Here, we report the first case of Sjögren's syndrome concurrent with protein‐losing gastroenteropathy in which secondary systemic capillary leak syndrome developed, together with a review of the literature.

## 2 CASE HISTORY

The patient was an 88‐year‐old man with a history of dyslipidemia, right corneal transplantation, and cataract surgery. One month prior to hospitalization, he experienced respiratory distress on exertion and pedal edema, for which he consulted his local physician. Upon receiving a diuretic, Chinese herbal medicine, and albumin drip infusion, his symptoms improved. One week prior to hospitalization, the patient experienced abdominal bloating and was referred to our hospital after being diagnosed with pleural effusion with ascites; he was subsequently hospitalized.

On admission, the patient's height was 155 cm and weight was 62.2 kg, and he presented with mild edema of the fingers and marked fast pitting edema in both legs. Laboratory tests on admission revealed hypoalbuminemia, with 2.8 g/dL Albumin (Table 1). However, as the urinary protein/creatinine ratio was 0.926 g/g Creatinine, nephrotic syndrome was ruled out.

**Table 1:** Blood tests results

	Initial
Hemoglobin (g/dL)	11.3
Hematocrit (%)	34.0
Mean corpuscular volume (fL)	85.9
Platelets (×10^9^/L)	254
Leukocytes (×10^9^/L)	6.7
Neutrophils (×10^9^/L)	3.5
Lymphocytes (×10^9^/L)	2.4
Prothrombin time ratio (INR)	1.03
D‐dimers (μg/mL)	3.8
Uric acid (mg/dL)	4.4
Blood urea nitrogen (mg/dL)	22
Creatinine (mg/dL)	0.75
Total protein (g/dL)	5.8
Albumin (g/dL)	2.8
Total bilirubin (mg/dL)	0.4
AST (IU/L)	28
ALT (IU/L)	11
Alkaline phosphatase (IU/L)	227
LDH (IU/L)	210
Total cholesterol (mg/dL)	211
Sodium (mEq/L)	133
Potassium (mEq/L)	3.9
Chloride (mEq/L)	102
Calcium (mg/dL)	8.2
Creatine phosphokinase (IU/L)	63
CRP (mg/dL)	2.3
TSH (μU/mL)	3.4
BNP (pg/mL)	88.2
Interleukin‐6 (pg/mL)	25.2
Vascular endothelial growth factor (pg/mL)	179
Gamma globulin (g/dL)	0.91
IgG4 (mg/dL)	48
Anti‐SS‐A antibody	Positive
Anti‐SS‐B antibody	Positive

As platelet count was normal and abdominal ultrasonography revealed no sign of liver cirrhosis, liver failure was also ruled out. Heart failure and hypothyroidism were also ruled out. Pleural effusin test revealed exudative, with 83 IU/L LDH and 3.5 g/dL protein, but it was modified according to infusing albumin by physician who treated him before the admission. So, actually pleural effusion seemed to be transudative.

Contrast‐enhanced computed tomography of the chest and abdomen revealed lymph node edema of <1 cm in the bilateral axilla and mediastinum.

A right axillary lymph node biopsy was normal and laboratory tests revealed mildly elevated levels of interleukin‐6 at 25.2 pg/mL, vascular endothelial growth factor (VEGF) at 179 pg/mL, gamma globulin at 0.91 g/dL, CRP at 2.3 mg/dL, and IgG4 at 48.0 mg/dL (Table 1); thus, Castleman disease was ruled out. Serum positivity for anti‐SS‐A and anti‐SS‐B antibodies (Table 1), as well as an ocular staining score of >4 points in both eyes, indicated that the subject satisfied 2 out of 3 items of the 2012 classification for Sjögren's syndrome established by the American College of Rheumatology and The Sjögren's International Collaborative Clinical Alliance; thus, the patient was diagnosed with Sjögren's syndrome.^2^ Furthermore, gum test revealed dry mouth, with a saliva flow rate of 0.5 mL/10 min.

Biopsies of the stomach, duodenum, rectum, skin, abdominal wall fat, and bone marrow were performed; however, since amyloid deposition was not observed, amyloidosis was ruled out. Biopsies of the stomach and duodenum showed only mild lymphangiectasis. Immunologic studies to assess complements were not performed.

**Figure 1:**
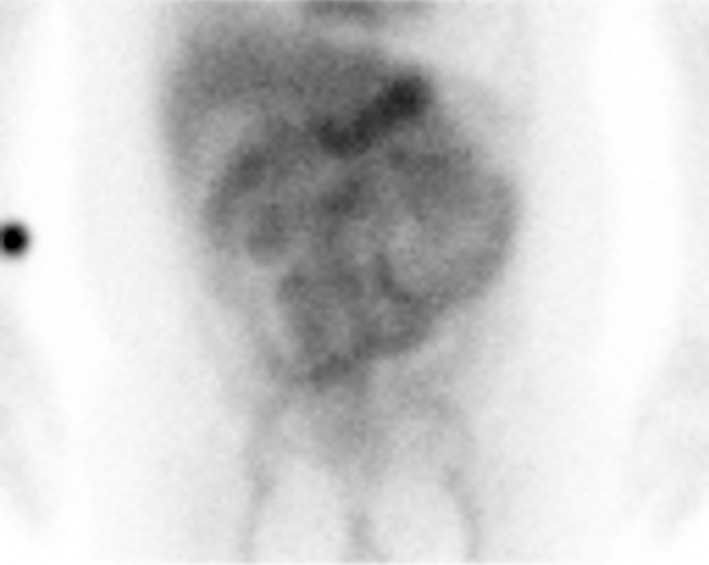
Technetium‐99 m‐labelled (^99m^Tc‐labelled) human serum albumin (HSA) scintigraphy. 2 minutes after intravaenous injection of ^99m^Tc‐labelled HSA, there was mild accumulation in the small bowel, and it became clear. 3 hours and 30 minutes after injection, there was movement of accumulation in the anus side. Typical features of protein losing gastroenteropathy were documented

Fecal fat staining was negative. However, technetium‐99 m‐labeled human serum albumin scintigraphy revealed early radioisotope accumulation in the small bowel (Figure 1). Because of this typical finding of protein‐losing gastroenteropathy and absence of gastrointestinal bleeding, we diagnosed protein‐losing gastroenteropathy.

Fecal alpha‐1‐antitrypsin test was useful but unavailable at our hospital.

## 3 OUTCOME

On day 18 of hospitalization, a dose of prednisolone at 30 mg/d (0.5 mg/kg/d) was initiated. On day 24, a sudden drop in blood pressure, reduced level of consciousness, elevated level of hematocrit (Ht) at 48.3%, pleural effusion with ascites, pericardial effusion, systemic edema and decreased level of serum albumin at 1.7 g/dL were observed. This led to a suspicion of protein leakage from a location other than the gastrointestinal tract, and upon diagnosis of secondary systemic capillary leak syndrome,^3^ human gamma globulin at a dose of 0.4 g/kg/d was administered for 5 days. Because the improvement in edema was poor, the dosage of prednisolone was increased to 50 mg/d starting on day 16; however, the patient showed little response to this increase in dosage of prednisolone. From day 28 to day 30, steroid pulse therapy was administered with methylprednisolone at 1 g/d and theophylline therapy was simultaneously initiated and maintained at a serum concentration of 10‐20 μg/mL. On day 31, his body weight reached its maximum value of 71.2 kg (9.0 kg increase since admission), and prednisolone was recommenced at 50 mg/day. From day 32 to day 42, 20% albumin at 100 mL/d and furosemide at 20 mg/d were administered. As a result, his body weight gradually decreased and the edema also improved. On day 45, theophylline was discontinued, and the dosage of prednisolone was reduced on days 46, 61, and 75 to 40 mg/d, 30 mg/d, and 25 mg/d, respectively. On day 80, he was discharged from the hospital, and he subsequently continues to receive treatment on an outpatient basis.

## 4 DISCUSSION

Autoimmume diseases that cause protein‐losing gastroenteropathy include scleroderma, systemic lupus erythematosus, Sjögren's syndrome, rheumatoid arthritis, mixed connective tissue disease, and dermatomyositis, and the cause of protein‐losing gastroenteropathy associated with autoimmune disease is thought to be related to capillary hyperpermeability.^1^ Among autoimmune diseases, there have been 21 published reports from 1988 to 2017 in English and Japanese of cases presenting with Sjögren's syndrome concurrent with protein‐losing gastroenteropathy, as in the present case (Table 2).^1^, ^4^, ^5^, ^6^, ^7^, ^8^, ^9^, ^10^, ^11^, ^12^, ^13^, ^14^, ^15^, ^16^, ^17^, ^18^, ^19^, ^20^, ^21^, ^22^, ^23^ Among such publications, there are no reports of secondary systemic capillary leak syndrome, thereby making our case the first case to be reported.

**Table 2:** The previous reports of PLGE patients associated with SS

Author	Publish year	Age	Sex	Nationality	Alb (g/dL)	SS‐A	SS‐B	ANA	Complication	Steroid	Other therapy	Outcome	Reference
Sugiyama T	1988	47	F	Japanese	1.6	+	‐	64	Chronic thyroiditis	PSL 60 mg (p.o.)		Improve	1, 4
Yamada H	1994	38	F	Japanese	2.3	ND	ND	ND	SLE	PSL		Improve	5
Iizuka H	1996	28	F	Japanese	1.4	ND	ND	ND	Chronic thyroiditis	PSL 40 mg		Improve	6
Inoue R	1996	49	F	Japanese	2.4	ND	ND	ND	n.p.	PSL 40 mg		Improve	7
Mok MY	1997	54	F	‐	1.4	ND	ND	ND	‐	PSL 60 mg (p.o.)	CPA 100 mg	Improve	8
Imai K	2002	64	F	Japanese	2.7	+	+	ND	RA	‐	Ubai‐en (Kampo medicine)	Improve	9
Hsieh TY (case1)	2002	37	F	Taiwanese	1.4	+	ND	320	n.p.	m‐PSL 750 mg × 3 d 2course (i.v.) + PSL 30 mg (p.o.)	HCQ 200 mg	Improve	10
Hsieh TY (case2)	2002	50	F	Taiwanese	1.1	+	ND	640	n.p.	m‐PSL 750 mg × 3 d 3course (i.v.) + PSL 30 mg (p.o.)	HCQ 200 mg	Improve	10
Choi HJ	2004	50	F	Korean	ND	ND	ND	ND	ND	PSL 60 mg (p.o.)		Improve	11
Ushiyama A	2004	61	F	Japanese	1.8	+	‐	320	Chronic thyroiditis	PSL 40 mg (i.v.)		Improve	12
Nagashima T	2009	41	M	Japanese	1.3	+	+	1280	n.p.	PSL 70 mg (i.v.) + m‐PSL 1 g × 3 d (i.v.)		Improve	13
Nasu T	2011	59	F	Japanese	2.8	+	‐	ND	RA, Chronic thyroiditis	PSL 50 mg (p.o.) + m‐PSL1 g × 3 d 2course (i.v.)	CPA pulse + MZR 150 mg	Improve	14
Uraoka Y	2012	42	F	Japanese	1.5	+	ND	ND	n.p.	m‐PSL 1000 mg×3 d (i.v.) + PSL 20 mg (p.o.)	CPA pulse + rituximab	Improve	15
Kakigao K	2012	58	F	Japanese	1.5	+	‐	ND	MCTD, hypothyroidism	PSL 45 mg (p.o.)		Improve	16
Chen HY	2013	69	F	Chinese	ND	+	+	ND	ND	PSL (p.o.) + m‐PSL (i.v.)		Improve	17
Yamashita H	2014	51	F	Japanese	1.5	+	+	2560	Interstitial pneumonia	PSL 60 mg (p.o.)		Improve	18
Liao CY	2015	30	F	Taiwanese	1.8	+	ND	5120	n.p.	PSL 30 mg (p.o.)	HCQ 400 mg	Improve	19
Gupta A	2015	58	F	White	2.6	+	+	1280	Type 1 RTA	PSL 60 mg (p.o.)	CPA 800 mg/mo		20
Izumi Y	2016	64	F	Japanese	3.0	+	‐	‐	n.p.	PSL 50 mg (p.o.) + m‐PSL 500 mg × 3 d (i.v.)	MZR 200 mg	Improve	21
Ofuji K	2016	73	M	Japanese	2.7	+	‐	80	Dermatomyositis	PSL 45 mg (p.o.)		Improve	22
Hadigal S	2017	67	M	United States	2.5	+	ND	640	Pleural effusion	PSL	HCQ	Improve	23
This case	2018	88	M	Japanese	2.8	+	+	40	n.p.	PSL 30 mg (p.o.) + m‐PSL 1000 mg (i.v.)	IVIG 20 g + theophylline	Improve	

PLGE, protein‐losing gastroenteropathy; SS, Sjögren's syndrome; SS‐A, Anti SS‐A antibody; SS‐B, Anti SS‐B antibody; ANA, Anti nuclear antibody; PSL, prednisolone, p.o., per os.; SLE, systemic lupus erythematosus; n.p., not particular; CPA, cyclophosphamide; ND, no data; RA, rheumatoid arthritis; m‐PSL, methylprednisolone; HCQ, hydroxychloroquine; MZR, mizoribine; IVIG, intravenous immunoglobulin; i.v., intravenous; MCTD, mixed connective tissue disease; RTA, renal tubular acidosis.

Among the 21 reported cases, 18 were females and 19 were reported from East Asia. The present case is also considered a rare case, as the patient is elderly and male. Among the 21 patients reported, 20 received prednisolone, and in the event of a poor response to steroids, additional treatment was administered (eg, cyclophosphamide, hydroxychloroquine, mizoribine, and rituximab), resulting in the alleviation of symptoms in all patients.

In the present case, a sudden drop in blood pressure and hemoconcentration were observed on day 24. Moreover, pleural effusion with ascites, pericardial effusion, and systemic edema were observed. Thus, protein leak other than that from the gastrointestinal tract was suspected, leading to the diagnosis of secondary systemic capillary leak syndrome.

Systemic capillary leak syndrome is a rare disease characterized by 3 features, comprising hypotension, hypoalbuminemia, and hemoconcentration, and is said to cause disruption of vascular endothelial cells, resulting in the leakage of plasma proteins into the interstitial compartment. Because of normal blood pressure and no sign of hemoconcentration on admission, systemic capillary leak syndrome did not present on admission.

The involvement of VEGF has been suggested in the extravascular leakage of protein associated with systemic capillary leak syndrome. Moreover, because our patient also presented with elevated levels of VEGF, VEGF appears to be involved. There has been a reported case of systemic capillary leak syndrome treated with high‐dose intravenous immunoglobulin therapy (IVIG), theophylline therapy (blood concentration at 15‐25 μg/mL), terbutaline (beta‐2 receptor agonist), anti‐human‐TNF‐alpha monoclonal antibodies, and anti‐VEGF antibodies.^3^

Although our patient was first treated with prednisolone at a dose of 30 mg/d for protein‐losing gastroenteropathy, he later developed secondary systemic capillary leak syndrome, and thus IVIG was administered at a dose of 0.4 g/kg for 5 days, a regimen that is also considered to be a valid treatment for Sjögren's syndrome. Upon completion of IVIG therapy, there was no clear therapeutic effect, and additional treatment with steroid pule therapy and theophylline therapy were administered, which successfully alleviated the symptoms. However, it is unclear whether the steroid, IVIG, or theophylline therapy was effective.

In conclusion, we report the case of a patient who presented with Sjögren's syndrome concurrent with protein‐losing gastroenteropathy and whose condition became severe upon developing secondary systemic capillary leak syndrome. However, the patient's condition was immediately identified, and he recovered with the simultaneous administration of treatment for Sjögren's syndrome and systemic capillary leak syndrome.

## CONSENT

While ensuring the anonymity of the patient, we obtained the patient's written consent to report his case.

## CONFLICT OF INTEREST

The authors have no conflicts of interest to declare with regard to this report.

## AUTHORSHIP

KW: played a key role in the patient's treatment and wrote the draft of the manuscript. SM: played a key role in the patient's treatment. All authors were involved in the treatment, preparation of the original manuscript, and revision of the original manuscript. They have agreed to hold accountability for the translation of this report.
